# Dialectical Thinking: A Proposed Foundation for a Post-modern Psychology

**DOI:** 10.3389/fpsyg.2022.710815

**Published:** 2022-06-13

**Authors:** Nikolay Veraksa, Michael Basseches, Angela Brandão

**Affiliations:** ^1^Faculty of Psychology, Lomonosov Moscow State University, Moscow, Russia; ^2^Department of Psychology, Suffolk University, Boston, MA, United States; ^3^Center for Philosophy of Sciences of the University of Lisbon, Lisbon, Portugal

**Keywords:** dialectical thinking, post-modern psychology, limits of universalistic-formal analyses, limits of relativistic analyses, psychotherapy integration, ontogeny

## Abstract

For the authors, the way from a modern to a post-modern psychology requires dialectical thinking. Dialectical thinking recognizes the importance of contradiction, change, and synthesis; it also includes recognition of the value as well as limits of modern epistemological approaches. The article describes foundations for both ongoing efforts to understand and research the ontogeny of dialectical thinking and for appreciating the scope of dialectical thinking and its relevance for establishing a bridge from modern to post-modern psychology.

## Introduction

The meanings of “modern” vs. “post-modern” is itself a topic for which several generations have contributed to an extensive literature. While space does not allow review of this literature, we could take as a jumping off point what could be considered a “popular” understanding as reflected on the internet “question-answering” website, Quora:

The essential **difference between modern and postmodern** [in any discipline or field] is that modernism [generally the period 1890s to 1945] reflects rational thought and logic whereas **postmodernism** [postwar, nebulous start date] rejects logic. In short, modernism is theoretical and objective; postmodernism is subjective (Quora, 2014, first answer in Google.com search for “modern vs. postmodern”).

From the authors’ perspective, this quote reflects a popular misunderstanding that what postmodernism rejects is rational thought and logic in general, whereas, we understand it to reject a particular and very limited conception of rational thought and logic that is indeed associated with the concept of objectivity.

We understand the *modern perspective* on inquiry as assuming its purpose is to make sense of the world, we experience by discovering (or, in a more sophisticated version, creating, models of fundamental fixed realities)—basic elements and immutable laws. The rational thought and logic that *postmodernism* rejects is the specific logic of inquiry that assumes that such basic elements and immutable laws objectively exist, prior to and independent of the inquiry that describes them.

A post-modern psychology may begin by acknowledging the impact of the subjectivity of inquirers. It also asserts that the assumption that basic elements and immutable laws exist prior to and independent of the inquiry is a premise of modernist inquiry, rather than a justifiable ontological or epistemological principle. But in our view, a post-modern psychology must proceed from there to an articulation of a view of rational thought and logic that:

- makes epistemological progress possible while taking into account the role of *subjectivity in processes of inquiry*, and,
- includes the possibility of studying:
  1. elements—as *changeable* and given meaning by the wholes/contexts of which they are parts, rather than as being fixed in nature and,
  2. laws and regularities—as forms of organization that organize relationships among elements and that *change and develop over time*.

Without such an expanded view of the rationality that offers a model for the construction of more intersubjective, epistemically adequate understandings over time, post-modern inquiry would be limited to the simple accumulations of descriptions of various subjective interpretations of phenomena.

Post-modern critique in the absence of articulation of a more complete and powerful view of rationality and logic leads to what we would call “relativism.” We view relativism as equally limited and epistemologically inadequate to the modernist approach of taking discovery of basic elements and immutable laws as the central goals of inquiry. Basseches’ book on dialectical thinking (1984), while documenting the development of the capacity for dialectical thinking in adulthood, also made the case that a dialectical understanding of the nature of inquiry, rationality, and logic may be understood as providing a more epistemologically adequate organization of inquiry than either modernism or relativism taken by itself, while drawing heavily on the contributions of both modernists and post-modernists in various fields, including psychology.

In the psychological study of dialectical thinking three traditions can be differentiated. For convenience, we refer to them in a somewhat oversimplified way as “Russian,” “Neo-Piagetian,” (e.g., United States, Canada, and Western Europe), and “Asian” (e.g., China, India, and Japan). The Russian and Neo-Piagetian approaches focus more on the ontogeny of dialectical thinking, while the Asian approach focuses more on tendencies associated with culture. In this article’s, overview of the development of dialectical thinking and its implications for psychology research and practice, we cite work in the Neo-Piagetian and Russian traditions. Here, we very briefly discuss the Asian approach, and why the article focuses on the other two.

The Asian approach is reflected in works by Nisbett, Peng (Peng and Nisbett, 1999) and Hamamura (Hamamura et al., 2008), among others. Peng et al. (2006) have studied a form of Eastern thought that they call naïve dialecticism and trace to East Asian Confucianism. It is characterized by the acceptance of contradiction and the idea of a constant flow or change in every aspect of the world, considering reality as a process (Spencer-Rodgers et al., 2010). This naïve dialecticism is characterized by a way of thinking that, contrary to Aristotelian logic, accepts oppositions and searches for a “middle way” or a compromise approach (Peng and Nisbett, 1999).

Account of naive dialecticism of Peng and Nisbett (1999) has been criticized by other researchers of Asian thought including Chan (2000) and Ho (2000). Ho criticized Peng and Nisbett for misunderstanding the relationships of contradiction with formal logic and dialectical thinking. Ho argues that formal logic applies to concrete problem analysis in a given moment, while dialectical thinking analyzes processes. Ho further states that Peng and Nisbett assume dialectical thinking is the cognitive ability to accept contradiction along with the “Confucian” non-dialectical tendency of Chinese to look for a compromise as a problem solution. Ho, following perspective of Basseches (1984), considers dialectical thinking as including metacognition that leads to revealing hidden implicit contradictions, striving to resolve them, and bringing the thinker to a more complex level of understanding of the problem.

We agree that Ho makes an important distinction and we propose the following points: First, naive dialecticism mainly represents a cultural style of thinking with its advantages and disadvantages, characterized by tolerance of contradictory beliefs (Hamamura et al., 2008; Ng and Hynie, 2016; Zheng, et al., 2021). Second, what has been called naive dialecticism can be described as a *dialogical relativism*. Naive dialecticism considers conflicting perspectives as belonging to the same whole within a process of constant change (i.e., it is dialogical). It thereby goes beyond the simpler perspective of relativism as description of various subjective interpretations of phenomena. It does not, however, specify any metacognitive capacities for discovering how to overcome contradictions thereby integrating the oppositions and creating new knowledge. Third, how to understand naive dialecticism in relation to the roots and forms of dialectical thinking found even in preschoolers by Russian psychologists (Shiyan, 2008; Bayanova, 2013; Veraksa et al., 2013), as well the “post-formal” organization of dialectical thinking studied by Basseches (1984) and his neo-Piagetian successors, are important questions for future study.

The work of each of the first two authors constitute contributions to understanding dialectical thinking as both a psychological phenomenon with a history in life-span human development, and as a systematic approach to inquiry that is applicable to a post-modern psychology. These two contributions can also be understood as extensions of two projects of the modern era which took shape in the early 20th century: (1) Jean Piaget’s effort to create a genetic epistemology by modeling and illustrating the basic developmental processes that underlie the creation of organizations of understanding and (2) Lev Vygotsky’s development of the dialectical point of view on psychological processes in the context of the historical tradition of dialectical analysis that took shape in the early 20th century in Russia. Both of these projects led to studies of dialectical thinking as a psychological phenomenon beginning in the 1970’s and 1980’s. We will summarize this work, and its relevance to establishment of a foundation for the development of a post-modern psychology.

## Dialectical Thinking in Piaget’s Work and Neo-Piagetian Research on Dialectical Thinking as a Psychological Phenomenon

The genetic psychology of Piaget (1952, 1954, 1970, 1967) used psychological research to address epistemological questions. It studies and describes both structure and process in cognitive ontogenesis. The structural aspects include descriptions of forms of cognitive organization corresponding to the well-known stages of sensorimotor structures, representational structures, concrete operational structures, and formal operational structures. In each case, the organizational basis of the structure’s stability is described, often in largely mathematical terms, and the features specified that make the structure more epistemically adequate than the ontogenetically prior organization—i.e., that enable the structure to deal with more complexity in more stable ways. But a clear tension can be identified between these *structural* aspects of Piaget’s theory and his *framework for conceptualizing the process of development* of cognition and knowledge. In the *process* theory, consecutive moments of assimilation of novel experience to existing structures, and accommodation of those structures to novel experience lead to “conflict” or “disequilibrium, which requires “equilibration”—the construction of new more integrative forms of organization, to resolve. Piaget’s *process* theory would surely be considered to be based on dialectical thinking using Basseches’ criteria (1978, 1984). “The cognitive structures *described by* Piaget’s theory could never account for the intellectual tools Piaget relied on *to create his theory*” is one way of expressing this tension. In this section, we will begin by discussing how Basseches (1984) addressed this tension.

Basseches (1978, 1984) began with an effort to describe dialectical thinking as an entirely different *form of organization of thought* that contrasted with the closed system form of organization of thought represented by Inhelder and Piaget’s description of formal operational thought (1958). At the same time, Basseches advanced the argument that dialectical thinking, employing a model of open systems, interacting with, changing, and potentially becoming integrated with each other over time, provided a greater degree of equilibrium (a fundamental criterion of epistemological adequacy for Piaget) than closed-system modeling taken by itself. This greater equilibrium could explain movements from formal to dialectical thinking in adults as they encountered limitations of or contradictions among closed-system models, as well as offering a more adequate foundation for systematic inquiry. Basseches had the intention of describing a dialectical form of organization of thought that in neo-Piagetian terms could be described as “post-formal,” implying hierarchical integration—i.e., that it could both make use of the power and value of formal analyses based on closed-system models, while at the same time being able to articulate the limits of formal analyses and transcend them. It could understand particular closed-system models as moments, within larger processes of contradiction and transformation—differentiation and integration. In address to the goals of this special issue, we are proposing that a post-modern psychology could similarly make use of analyses based on closed-system models, while at the same time using dialectical analyses to understand and transcend the limits of those closed-system analyses.

Thus, for understanding how dialectical analysis transcends the limits of closed-system analysis it is important to keep in mind the related Piagetian core concepts of *equilibrium* and *equilibration*. The cognitive concept of equilibrium, as the analogous biological concept of homeostasis, represents the capacity of the organism or system to maintain stability by adjusting to internal or external conditions that change the prior state. From the Piagetian genetic epistemological viewpoint, change represents development if, and only if, a reorganization of activity happened in such a way that, a new organization becomes capable of assimilating a greater variety of experiences, while maintaining stability in its core organizational features. The result of change in this case will be a cognitive organization with a *higher level of equilibrium*. Basseches (1984) argued that specific analyses as well as general approaches to inquiry based on the organizing principle of dialectic (which integrates dimensions of contradiction, change and system-transformation over time), will be more epistemically adequate than analyses and approaches to inquiry based on the formal operational principle of the closed system of lawful relationships of elements (Inhelder and Piaget, 1958).

Basseches (1984) addressed the following four questions, (1) “What is a dialectic?” (2) “How does a dialectical analysis, based on a model of dialectic, differ from a formal analysis, based on a model of a closed-system?” (3) “What gives dialectical analyses greater equilibrational/adaptive power” than formal analyses?” and (4) How can one identify, in examples of adult thought, the use of a dialectical model as well as formal models?”

Basseches’ research used a sort of bootstrapping method. He started with what he recognized as dialectical analyses from intellectual history. He included a wide range of dialectical analyses of very different content that came from various intellectual disciplines with the intent of understanding *the underlying model of dialectic that they shared in common*.

Common features of these analyses were used both to derive a definition of dialectic and to recognize common patterns of underlying models as well as schemata or “moves-in-thought” that constituted “family resemblances” across this wide range of dialectical analyses that could also be recognized within the intellectual development of adults (Basseches, 1980, 1984). In his review of analyses from intellectual history, Basseches also recognized “dialectical” approaches, as well as “universalistic formal” and “relativistic” ones, as representing three alternative sets of “styles of inquiry,” “intellectual sensibilities,” and “world outlooks.” The latter two sensibilities seemed to understand the goals of inquiry in radically different ways, while the dialectical sensibility understood the goals of inquiry in a third way, which both incorporated aspects of the universalistic and relativistic approaches and yet transcended both.^1^

### Three Styles of Inquiry: Universalistic Formal, Relativistic, and Dialectical

Basseches (1984) characterized the *universalist formal approach* to inquiry by the assumptions that there is a “universal order to things” which is the foundation for establishing “fixed universal truths.” This order lends itself to description in “an abstract and formal way” and “all manner of phenomena in the universe may be found to fit in their places within this order.” Systematic inquiry, whether scientific or philosophical, is aimed at describing this order. The sentiments associated with this approach are positive ones toward powerful abstract systems of ordering which capture the commonality or relationships among apparently different things.” A good example of a system which was greeted with great enthusiasm is Chomsky’s (1957) work in linguistics. Chomsky described linguistic structures which he claimed to be at the core of all the languages of human speakers all over the world, regardless of the many phenotypical differences among human languages. Universalistic formalists tend to have negative sentiments toward relativistic reasoning, which they view as “accepting too much sloppiness or disorder in the workings of the universe.” They sometimes even view relativistic reasoning “as sloppy thinking which has retreated from the task of imposing strict order on everything” with an implication that this retreat might be due to “laziness” or “lack of intellectual power” (Basseches, 1984, p. 10).

In contrast, Basseches wrote that the *relativistic approach* to inquiry assumes (1) “that there is not one universal order to things but … many orders…” and (2) “that different individuals, groups, or cultures order reality in different and incompatible ways. Thus, order in the universe is entirely relative to the people doing the ordering.” Systematic inquiry, whether scientific or philosophical, is aimed at description, appreciation, and even creation of “as wide a range of different orderings as may exist and be interesting and useful.” Appreciation of diversity is a common positive sentiment among relativists. They greet with enthusiasm work that shows how things can be looked at in a variety of ways. Two such examples are “anthropologists’ ethnographies of distant cultures (e.g., Mead, 1928)” and “idiographic approaches in personality psychology” (e.g., Allport, 1937). Relativists also value “tolerance or mutual appreciation among people who order the universe in different ways.” Relativists tend to have negative sentiments toward “what they perceive as imperialism, including intellectual imperialism.” It is often seen as imperialistic (1) “when universalistic formalists claim that one way of ordering things is *the right way*, equally applicable to phenomena experienced by all persons, groups, and cultures,” or (2) “when universalists create schemes which acknowledge diversity of orderings but then order these diversities themselves within some over-arching framework that imputes greater value to some orderings than others.” Anthropological theories which treat some cultures’ modes of ordering as “primitive,” or personality psychologists who treat some individuals as “pathological,” using “standards taken from the anthropologists’ own ‘civilized’ culture” or the mental health community’s culturally shared consensus of what is “healthy” respectively, are frequently seen as equally imperialistic to viewing one framework as universally valid. In sum, any view which claims that one person’s way of viewing things is truer or better than another’s is regarded with distrust if not hostility by relativists except perhaps relativists’ own view that their way of “conducting inquiry is better than that of the universalists” (Basseches, 1984, p. 10–11).

Basseches characterized the *dialectical approach* as charting a third alternative course. This approach assumes that “the evolution of order in the universe is an ongoing process” and that the *process of finding and creating order* in the universe is fundamental to human life and inquiry. “Thus, systematic inquiry, whether scientific or philosophical, is aimed at contributing to this process, and it is the process itself for which dialectical thinkers’ most positive sentiments are reserved.” Therefore, dialectical thinkers tend to “regard positively that which contributes to these processes and negatively that which obstructs them.” The process of creating order is understood “as occurring through efforts to discover what is left out of existing ways of ordering the universe, and then to create new orderings which embrace and include what was previously excluded.” Basseches continued:

Dialectical thinkers can therefore be expected to share with universalistic formalists the negative reaction to relativistic reasoning, when the latter seems simply to acknowledge difference and disorder, and to retreat from efforts to find and create more powerful orderings. At the same time dialectical thinkers would share with relativists the reaction that it is dangerous to believe that an all-inclusive ordering is possible. For it is precisely when one thinks one has a achieved such an ordering that one stops actively looking for what is left out and what is different and in fact, (universalistic formalists) start to systematically defend (themselves) against perceiving such phenomena, (at which point inquiry may become limited to the extension of previous structures of ordering to newly investigated phenomena). [Basseches, 1984, p. 11].

Basseches goes on to comment on the issue of imperialism from the perspective of dialectical inquiry:

Imperialism forces a way of life on others making it less likely that their own preferred way of life will be expressed. Intellectual imperialism imposes an order on the lives and meanings of others, making it less likely that the orderings created by others will be perceived. The easing up on the quest to find difference and disorder disrupts the fundamental process of inquiry as much as does the easing up on the effort to try to create order and unity when disorder and differences are discovered (Basseches, 1984, p. 11).

To end his characterization of a dialectical orientation toward inquiry, Basseches added that both types of work most appreciated by universalists and relativists are also valued by dialectical thinkers. However, the core of the dialectical approach is the locating of both types of work within an ongoing process of differentiation and integration of knowledge which transcends the limits of each approach to inquiry, taken by itself. We note that this very way of characterizing the three approaches represents a differentiation of universalistic formal from relativistic approaches to inquiry, and that the equilibrative power of dialectical thinking and dialectical analyses is demonstrated in the integration of the two previously differentiated approaches. Our argument in this paper is one for similarly differentiating modern approaches to psychology from approaches of post-modern psychology that appear to trade the acceptance of the role of subjectivity for a rejection of rationality, and then basing the further development of post-modern psychology on an integration of these two differentiated components. Later in this article, we will consider the nature of dialectical analyses, and the way they represent products of a dialectical approach to inquiry.

Returning to Basseches’ research, the next step after a description of the implicit model that organizes dialectical thinking and the component schemata by which dialectical thinking could be recognized was as follows. Pilot interviews were conducted with individuals about matters important to them to see if the previously identified schemata and underlying models derived from intellectual history could be found in the spontaneous thinking of individual adults, thereby identifying dialectical thinking as a psychological phenomenon. In these interviews, an adaptation of Piagetian “clinical method” (Flavell, 1967, p. 47) was used to probe the limits of participants’ capacities for cognitive organization. Based on these pilot interviews, the idea of dialectic was further clarified and the list of dialectical schemata recognizable in interviews was expanded. In the final phase, open-ended interviews related to the topic of the nature of education with the same structure of questions and the same “clinical method” interview techniques of probing were conducted with nine first-year students, nine fourth year students, and nine faculty members of the same highly selective liberal arts college. On the basis of these interviews, a measure of the development of the organizational form of dialectical thinking in an individual was developed and used to compare the cognitive approaches of members of all three subgroups.

Basseches (1984, p. 22) offered the following definition in answer to his first question, “What is (a) dialectic?”: “*Dialectic is developmental transformation* (i.e., *developmental movement through forms*) *which occurs via constitutive and interactive relationships*.”

To illustrate the components of this definition, we consider the forms of organization of theory and research on reproductive processes of pupfish (*Cyprinodon pecosensis*; Kodric-Brown, 1986). The phrase in the definition, “movement *through* forms” is meant to distinguish such movement from movement *within* forms. According to this research, when there is ample territory for nesting space habitat and substrate conditions are good, the reproductive activity is organized by dominance hierarchy. Thus, the activities of individual fish can be understood as movement *within* the form of the dominance hierarchy. As a reproductive season approaches, the mature males, who develop a bright blue color, engage in activities which establish their places in a dominance hierarchy. Then, in order of the hierarchy from top to bottom, each male chooses a “territory” which he tries to make as attractive as possible to welcome female pupfish to lay eggs. The females, who have a much more neutral color, in an order mainly organized by size (largest first), explore males’ territories and choose males’ territories in which to lay eggs. Males fertilize the eggs in their territories where they are protected until the baby fish are born.

In contrast, a movement *through* forms—a dialectic, occurs when due to some combination of the activity of the pupfish and other influences on their environment (seen dialectically as in *constitutive and interactive relationships*—to be elaborated below), a scarcity of territory suitable for nesting arises. This leads to the emergence of a more differentiated form of social organization in which males divide into three types, differentiated by reproductive strategy and appearance. The larger males become highly territorial, trying to create and defend their territories, while the smaller males become either “satellites” or “sneak-spawners.” The satellites are smaller in size than males with territories but have similar phenotypical appearances. They function as parasites on the territories, and they reproduce by disrupting and stalling copulation by the territorial males and managing to fertilize a few eggs themselves. In contrast, the sneak-spawners become more like females both behaviorally and in their phenotypical appearance, remaining closer to females in size and not having a blue color. To other males, they appear to be females passing through territories, while deciding where to lay eggs; but at the same time they take advantage of the opportunity to fertilize the eggs of the “true females” who have laid them. Thus the movement from a form of organization based on (1) sexual dimorphism, (2) similar reproductive strategies among males, and (3) dominance hierarchies, to one based on (1) “multimorphism,” (2) differences among males’ reproductive strategies, and (3) territoriality among only larger males, is an example of a dialectical movement *through* forms. To refer to this transformation as “developmental” implies that there is a direction to it—a direction associated with greater complexity and increased species-capacity for adaptation.

The definition of *dialectic* states that the development transformation occurs *via constitutive* and *interactive* relationships. The adjective “constitutive” means that the relationships play a role in the making the parties what they are. The adjective “interactive” implies that a relationship is not static but is characterized by motion or action of the parties upon each other. We may use the same example to illustrate these concepts. *Constitutive and interactive relationships* can be identified among the individual pupfish and between the group of pupfish and their environment. An individual pupfish’s activity is organized or *constituted* by its relationships with other pupfish as is clear when we say that a pupfish acts in accordance with its role in a dominance hierarchy. Role in a dominance hierarchy is not a characteristic of the pupfish as a separate entity, but a characteristic of that pupfish’s relationship to other pupfish. When we characterize an environment as “providing ample territory and an adequate substrate” for the reproduction of all the pupfish in it, we are not describing the environment in terms that frame it as prior to or separate from its relationship with pupfish, but rather in terms that depend on or are *constituted* by its relationship with pupfish.

The relationships among the pupfish and between the pupfish and their environment is interactive, as well as constitutive, in that the behavior of other pupfish affect the reproductive approach/activity and relative reproductive success for any given pupfish. Also, the activity of pupfish (e.g., creating overpopulation) can make a formerly adequate reproductive environment no longer adequate, while changes in the environment brought about by other factors than the pupfish’ activity, can also render the environment no longer adequate, leading to transformation of the social organization of the pupfish.

To summarize how the whole process described above provides an illustration of *dialectic*, the *interaction* within the *constitutive relationships* of pupfish and their environments generates a limitation to the viability of the earlier *form* of reproductive activity, which in turn results in a *developmental transformation* (*movement through forms*) to a new, more complex *form* of reproductive activity. Thus the entire process described above can be seen as an example of *dialectic*.

The next steps for Basseches were (1) to illustrate the differences between “dialectical analyses”—based on using the model of dialectic described above to understand all matter of phenomena, and “formal analyses”—based on using the model of a closed system of lawful relationships described by Piaget to understand phenomena, as well as (2) to show the greater equilibrative power of dialectical analyses. While formal analyses can be understood as efforts to identify and describe fundamental unchanging laws, dialectical analyses seek to identify and describe fundamental processes of change and the dynamic relationships through which such change occurs. Reflecting Basseches’ intent to derive from intellectual history an approach to understanding cognitive development in the lives of individual adults, he provided illustrations of the nature and power of dialectical analyses in both spheres.

While acknowledging the potential utility of the formal analyses, he tried to illustrate the power of dialectical analyses by showing the boundary conditions to which the scope of the value of formal analyses is limited. We recommend a similar approach to the creation of a post-modern psychology—demonstrating how the value of products of modern psychology is limited in each case to specific boundary conditions and proposing dialectical analyses and efforts at integration as the process of transcending those limitations.

Regarding intellectual history, Basseches (1984) wrote:

Dialectical analyses can be found in the history of a wide range of intellectual disciplines, representing the natural sciences (Provine, 1971; Feyerabend, 1975; Horz et al., 1980), social sciences (Jay, 1973; Mandel, 1973; Kilminster, 1979), and humanities (Jameson, 1971; Adorno and Horkheimer, 1979).^2^ They have been used to support political stances ranging from the very conservative (Hegel, 1821/1965) to the revolutionary (Marx and Engels, 1848/1955).

Two examples of dialectical analyses that are relatively well-known across academic fields, are:

1. Marx’s analysis (1844/1967) of the history of human productive and reproductive activity as a dialectical process in which (a) many aspects of economic, social, technical, and intellectual life are all interrelated within a form of organization of modes and social relations of production, and (b) tensions develop within these interrelationships and ultimately lead to a new form of organization of productive and reproductive activity (e.g., the replacement of feudal organization by capitalist organization); and
2. Kuhn’s dialectical analysis (1970) of the history of science in which the central ideas are (a) that research is shaped by dominant paradigms, (b) while paradigms make assumptions which serve as foundational premises for research, central to paradigms are pieces of insight-yielding research which can serve as a model for other researchers to follow, (c) in following paradigms subsequent research produces “anomalies” which are not easily reconciled with other extant knowledge, which in turn create discomfort among scientists, (d) while some scientists create and try to support *ad hoc* theories that preserve the dominant paradigms within increasingly unwieldy organizations of knowledge, other scientists start to create alternative paradigms which compete for followers with the dominant paradigm, (e) when a new paradigm (including assumptions, methodology, and ways of defining research problems and research solutions) attracts enough followers to represent a new dominant paradigm and redefine the nature of the field, a scientific revolution can be said to occur, and (f) While “normal science” guided by a paradigm can be characterized as puzzle-solving (corresponding to movement within forms in the definition of dialecticitc), the creation of new paradigms can be characterized as revolutionary (corresponding to transformational movement in the definition of dialectic).

Formal analyses in classical economic theory and philosophy of science respectively formed the backdrop against which dialectical analyses of Marx (1844/1967) and Kuhn (1970) were introduced as alternatives. Such formal analyses were characterized by assuming universally applicable laws—laws of economic behavior in one case and rules of evidence for hypothesis-testing in the other. The constitution of economic laws by existing relations of production and the possibility of transformation to new modes of production in which economic behavior would not follow the articulated laws were beyond the boundaries of scope of these formal analyses in the case of classical economic theory (Smith, 1776/1937). The constitution of rules of evidence by paradigms dominant in particular scientific communities and possibilities of scientific revolutions in which new paradigms would bring new rules of evidence were beyond the boundaries of the scope of formal analyses provided by confirmationist (e.g., Reichenbach, 1938) and falsificationist (e.g., Popper, 1959) philosophies of science.

Basseches’ goal of describing a post-formal organization of dialectical thinking, which integrates the abilities to use and yet transcend the limitations of formal analyses can be seen in the examples of dialectical analyses from intellectual history and adult development. Marxist economic theory integrates using classical theory to understand laws of economic behavior under capitalism with analyzing limitations and actual and possible transformation of those laws. Kuhnian analysis can use philosophical analyses to clarify rules of evidence which currently organize a discipline while simultaneously analyzing historically how current paradigms achieved hegemony and where they may confront their limits. Basseches provided several examples of typical challenges of adult life for which he contrasted the use of dialectical analysis with formal analysis. In these examples, he also considers “relativism” as an approach to analysis, as the relationships between relativistic thinking and formalistic and dialectical thinking respectively is another matter systematically considered in the book that is quite relevant to the topic of creation of a post-modern psychology.

The neo-Piagetian dimension of Basseches approach is reaffirmed when he juxtaposes psychological and epistemological perspectives after acknowledging that in questioning limitations and boundary conditions of individuals’ and communities’ assumptions, dialectical thinking trades off a degree of intellectual security for freedom from imposing limitations on self, communities, or outsiders. He asserts that “from the point of view of humanity, as an epistemic subject involved in an ongoing pursuit of truth, the added power made possible by the capacity for dialectical analysis seems important to recognize.” He ends with the claim that “dialectical thinking is an important psychological phenomenon, and that the capacity of dialectical thinking is an epistemologically important psychological attribute” (Basseches, 1984, p. 30).

Part II of *Dialectical thinking and adult development* (Basseches, 1984) presents his empirical work which addressed the fourth question, “How can one identify, in examples of adult thought, the use of a dialectical model as well as formal models.” Although dialectical thinking is defined by an assumed underlying model of dialectic, dialectical thinking is identified by instances of schemata, or patterned movements-in-thought which dialectical thinkers tend to make. Over the course of pilot research, Basseches described 24 such patterned movements-in-thought that could be identified in interviews. When clear instances of a sufficient proportion of these schemata were observed, including the most complex schemata, the inference was made that the subjects’ thinking was organized by a model of dialectic.^3^

In sum, Piaget’s description of the equilibration process, in which assimilation and accommodation led to moments of disequilibrium, which were then resolved through the creation of more stable forms of cognitive organization, was at the core of his association between equilibrative power and epistemic adequacy. This concept could be applied both to the ontogeny and phylogeny of intelligence. In both cases, encounters with the limitations of prior forms of organization are what leads to more integrative forms of organization within which the capacities of earlier forms of organization were retained. But Piaget only began to treat dialectical thinking as an object of investigation in his later years (Piaget, 1974, 1977). Basseches, also in the 1970’s, began work to extend Piaget’s developmental and epistemological theory by describing dialectical thinking as a form of organization of thought that provided greater equilibrium than the most developed cognitive structure that Piaget had described, “formal operations.” He claimed that while formal operations thinking relied on the idea of closed systems of lawful relationships in modeling various phenomena, dialectical thinking, with its application of the concept of developmental transformation over time, could articulate the boundary conditions for the utility of every closed-system model, and the processes by which those stability-focused models could become integrated into historical models of developmental transformation over time and the expansion of intersubjectivity that could occur with time. This idea is one pillar of our claim that dialectical thinking must be central to the most epistemically adequate approach to psychological inquiry.

In comparison with Piaget, the work of Lev Vygotsky was more explicit about the importance of dialectic in psychological inquiry from its outset. But it was left to those “neo-Vygotskians” who followed him, including Nikolay Veraksa, to begin an inquiry into dialectical thinking as a developmental psychological phenomenon. We discuss some of that work in the next section.

## Russian Research on Dialectical Thinking as a Psychological Phenomenon

Research on dialectical cognition in Russian psychology is closely connected to the work of Lev Vygotsky, who asserted himself as a dialectician and stated that “all true scientific thinking moves along the path of dialectics” (Vygotsky, 1983, p. 37). From a post-modern perspective, we wince at such broad claims to truth, and we recognize a variety of forms of scientific thinking and inquiry. But seeing neither modernist nor relativistic forms of inquiry as adequate for a post-modern psychology, we affirm the importance of understanding the alternative “path of dialectics” which Vygotsky followed.

Vygotsky’s emphasis on dialectical psychology was reinforced and further differentiated during Soviet times. Russian philosophy produced two concepts of dialectical cognition, which formed the context for further studies. One of them was based on the interpretation of *dialectical logic as a process* of understanding and dealing with evolving content, and the other stemmed from the analysis of *dialectical logic as a system of special operations and forms*.

The study of dialectical cognition, based on the latter understanding of dialectical logic, was started in the 1980s (Veraksa, 1981). The main distinguishing feature of this system that differentiates it from modern logic is that while modern logic perceives material or conceptual objects as basic fixed elements that are lawfully related to each other, dialectical logic treats such objects as *transformable structures*. Dialectical interpretation of these structures sees them as comprising relationships of opposites and allows the possibility that changes in such “internal” relationships of opposites can lead to changes in the structure of any such object. Studying the operation and development of the tools of this system of dialectical logic formed the basis of a research area that came to be known in Russia as “structural-dialectical developmental psychology” (Veraksa, 2006; Bayanova, 2013).

The *dialectical logic as a special system of logical tools* approach and the *dialectical logic as a process of understanding and dealing with evolving content* approach differed from each other not only in the foci and areas of their research. They also differed in the way their research was practically applied.

The analysis of Vygotsky’s works reveals that to a high extent it was dedicated to the development of a dialectical psychology and the application of a dialectical approach to the psychological problems of dialectical method. Yet he did not see it as viable to use the dialectical method directly adopted from philosophy:

(…) no psychological system can directly dominate psychology as a science without the help of methodology, that is, without creating a general science. The only legitimate adaptation of Marxism to psychology would be the creation of a general psychology with its concepts formulated in direct relation to general dialectics; that is, the dialectics of psychology. Any application of Marxism to psychology that follows other paths will inevitably lead to scholastic, verbal constructions, to dissolving dialectics in questionnaires and tests, to reasoning about things on the bases of external, casual, secondary features, to losing any objective criteria, to trying to negate any historical trend in the development of psychology, to a terminological revolution, in short, to a coarse deformation of Marxism and psychology. (…) in need of an as yet undeveloped but inevitable theory of biological materialism and psychological materialism as an intermediate science, which explains the concrete application of the abstract theses of dialectical materialism to the given field of phenomena.

Dialectics covers nature, thinking, history—it is the most general, maximally universal science. The theory of the psychological materialism or dialectics of psychology is what I called general psychology (Vygotsky, 1982, p. 419–420).

Vygotsky’s words cited above prove that he indeed set a goal to create such a scientific psychological theory that would mediate general dialectics and psychology as an independent science. He clarified:

(…) dialectics of psychology—this is what we may now call the general psychology (…) is the science of the most general forms of movement (in the form of behavior and knowledge of this movement), i.e., the dialectics of psychology is at the same time the dialectics of a man as the object of psychology, just as the dialectics of the natural sciences is at the same time the dialectics of nature (Vygotsky, 1982, p. 322).

From our point of view, Vygotsky at the same time (a) engages with the extant tensions in “content” that are essential parts of the evolution of that content while he (b) starts to represent the more “abstract” regularities in the dialectical form of such evolution across many different processes of content evolution. This allowed describing relatively stable moments in the development of any area of inquiry or any individual’s cognition in terms of the oppositions integrated in those moments. It also allowed the construction of the space of opportunities for a developing entity and a certain anticipation of possible conflicts and transformations at relatively stable and unstable moments in the course of its development.

We view Vygotsky as a dialectical constructivist who applied juxtaposition also in the framework of the analysis of the history of psychology:

The development of scientific ideas and views is accomplished dialectically. Opposite points of view on the same subject replace one another in the process of developing scientific knowledge, and a new theory is often not a direct continuation of the previous one, but its dialectical negation (Vygotsky, 1982, p. 201).

Rubinstein (1957); Davydov (1972), and Leontiev (1983), among other psychologists and philosophers, emphasized the importance of using a dialectical materialistic analysis in psychological research. The question of this possible application of the dialectical method implied the development of a concept of dialectical cognition, as an antithesis, in tension with traditional formal thinking. In Russian (Soviet) philosophy, this question was given much prominence in works of Ilyenkov (1974, 1979). In particular, in his work “Dialectical Logic,” he claimed that dialectical logic differed from the formal analysis of phenomena, the main difference being that dialectical logic dealt with contradiction.

The dialectical logic is indeed the opposite for the formal logic, but not in the sense that dialectical logic rejects all the conclusions of the formal logic…. The main goal of formal logic is to discover general logical forms, laws, and rules independently from their particular content.

Meanwhile, dialectical logic when solving the problem of truth at large, cannot be distracted from the concrete content of concepts, judgements, and inferences throughout the whole process of cognitive thinking (Andreev, 1985, p. 152–154).

Thus, the delineation is quite clear: the distinctive feature of formal logic became the operation of mental forms that were abstracted from their content, while dialectical logic began to be construed to be the logic of handling contradictions in the developing content.

It was argued that if dialectical logic existed, then it needed to possess the same formalism as formal logic did. In this case, it was to have different system operations (Maltsev, 1964). This opened up a second possibility of interpreting dialectics as the use of a “special logic” that differed from formal logic, and this circumstance could serve as a basis of dialectical cognition.

That created yet another perspective of dialectics as a special dialectical formal logic, both abstracted from its content and different from the traditional formal logic. This vision could lay the foundation for the construction of a formal theory of dialectical cognition, but only under a certain condition. That would be the description of abstract dialectical forms without material content. Therefore, such a description seemed basically unfeasible. This issue could be solved only if some unique dialectical operations were discovered, different from the traditional formal operations. Another obstacle was that almost all authors of that time acknowledged that dialectics comprised not only the analysis of development but also the analysis of the *emergence of new syntheses* within that process. Therefore, formalization of dialectics implied the formalization of the description of the process of development and the emergence of new syntheses. That, in its turn, looked totally impossible.

Many dialecticians agreed with that conclusion (Kopnin, 1973; Ilyenkov, 1979; Porus, 1979; Andreev, 1985, etc.).

Ilyenkov in particular encouraged:

[The use of conceptual dialectical categories] (…) not as terms or catchphrases, but as forms of thinking, as active logical forms of study of objective reality. And first of all, the *category of contradiction* in its strictly objective definitions, which, being reflected in scientific consciousness of people and time-proved throughout centuries of their practical use actually are *logical* definitions of that category—unlike the ones given in mathematical logic where a contradiction is a synonym for “zero truth,” where it is a synonym of “misperception” and “lie.” In regards to the formal derivation of some combinations (“conjunctions”) of signs from other combinations, these definitions are true, but they have nothing to do with thinking. Therefore, they cannot be called *logical* definitions of this concept (Ilyenkov, 1979, p. 143; words between brackets are those of the current authors).

Thus, Russian philosophy produced two different concepts of dialectical cognition—*dialectical logic as a process* of handling the evolving content and *dialectical logic as a system* of special operations and forms. It was in the context of these different concepts that further studies were conducted.

The former concept can be referred to as more “substantive,” as dialectical analysis cannot be separated from the substantive content that is evolving. The understanding of dialectical cognition as handling evolving content was further developed by Davydov (1972), who relied heavily on Ilyenkov’s work. Davydov considered dialectical cognition as a kind of thinking that analyzed the development of an entity based on its inner contradiction. In order to unfold the thought process in each specific analysis, it was necessary to find the initial key relation (contradiction) that gave rise to the whole variety of content as it formed. It is clear that the specific content presupposed the presence of a unique initial relationship, for which it was impossible to create a productive formal abstract analysis that disregarded the meaningful context in which the relationship formed and developed.

Thus, the approach represented by Davydov recognizes dialectical thinking in dialectical analyses that begin with the articulation of a specific core, which is generated by the initial contradiction and rooted in the conditions of a specific historical context and moment. It also traces out its sequelae over time from that key initial moment. As to the understanding of the mental or cognitive processes and capacities underlying an individual’s creating of such an analysis, two specific features of dialectical cognition should be considered: (1) an all-round vision of the development of reality in its motion, and (2) avoiding abstracting from the content.

The study of dialectical cognition, based on the understanding of dialectical logic as a special kind of formal logic, was started in the 1980s (Veraksa, 1981). One of the problems to be solved in the course of these studies was to show the presence of formal dialectical operations that differed from those of formal logic. Since dialectical operations were to represent elements constituting and being constituted by a logical structure, the structure would need to be describable in mathematical terms in the same manner as it was done in the works of Jean Piaget.

The problem was solved in two stages. The first stage was associated with a search for some abstract units to be used for the analysis of dialectical cognition. Relations of opposition were chosen as such. They were construed as including any content or fragments of content that could be in opposition to one another and be in a relationship of mutual exclusion. Coming up with the idea of variability of the relationship where the opposites can find themselves in different situations became the major breakthrough in the construction of dialectical logic and the understanding of dialectical cognition.

Veraksa and his colleagues found various examples in which opposites could be in different relationships: those of *transformation* (when one opposite is transformed into another); those of *transition* (when the transformation of one opposite into another does not occur immediately, but gradually, through an intermediate link); those of *conversion* (when the opposites pass into each other and then back); those of *mediation* (when for the two opposites are placed in such a situation where they act as components of the whole), etc (Veraksa, 2006).

The presence of such relationships between opposites apart from the mutual concordance and mutual exclusion allowed different consideration of the processes of object transformations. That required in the first place an identification of the characteristics that could be interpreted as opposites. Once they were ascertained, they could be matched with the schemes of previously discovered relationships which actually represented the options for possible transformations. In this case cognitive process unfolded on two levels: the formal abstract one (as handling the opposites) and on a concrete conceptual one (transition from the opposites to the content behind them).

Transformations described in such fashion could represent both the *real transformations of material objects* and *mental transformations of conceptual objects*. The former could be understood as processes occurring in inanimate and animate nature and the latter as dialectical mental acts. Obtained results in the form of descriptions of transformations of real and mental objects allowed linking them into a single logical structure.

**Figure 1:**
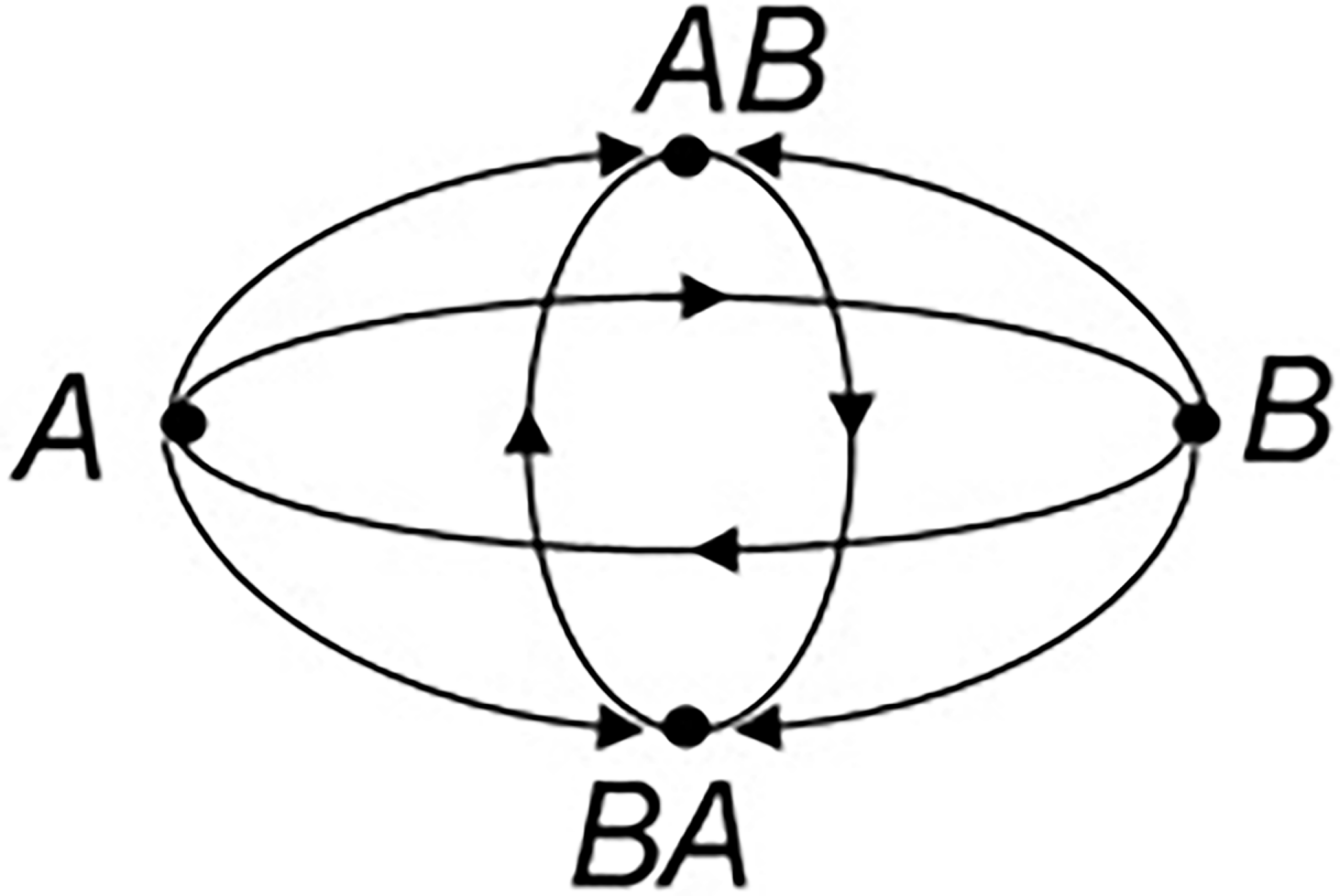
The structure of dialectical cycle *D*_2_.

The second stage was focused on the construction of a mathematical model of dialectical transformations (Veraksa and Zadadaev, 2012). Dialectical transformations were united into a dialectical structure D_n_, a structure that was built using discrete mathematical tools. *D₂* category became its elementary variant and was called a dialectical cycle. This cycle as a structure of dialectical logic has two extreme states that are in a relationship of opposition, and two opposite mediating states. If we denote the opposites through A and B, and their intermediate mediating states through AB and BA, then the simplest fragment of the dialectical structure can be illustrated with the help of Figure 1.

In this figure, the arrows denote the relationships of opposition and mediation. *The dialectical cycle differs from the traditionally understood dialectical triad of Thesis-Antithesis-Synthesis*. Of course, the dialectical cycle could be reduced by consecutive construction of triadic relations. However, there exist such objects and concepts that cannot be adequately described but with the help of a dialectical cyclic structure.

The structural-dialectical perspective sees the process of dialectical cognition as understanding of the transformations of the initial situation. First these transformations are represented in the conceptual plane, and then they appear as dialectical mental actions handling the relations of oppositions established by the subject for this particular situation (Veraksa, 2006). In connection with the above, *dialectical cognition is understood as a solution to a dialectical problem*. A dialectical task determines mental transformations performed by the subject.

First and foremost, we distinguish the action of a dialectical transformation among other dialectical mental actions. Its goal is to consider an object as its opposite. For example, Vygotsky (1983, p. 41), applied this dialectical action to the problem of defect: “A defect is not only weakness, but also strength. In this psychological truth is the alpha and omega of social education of children with disabilities.”

Discovering the opposites and their relationship by means of dialectical cognitive actions became the foundation of structural-dialectical analytical method. Application of this method proves that an individual is indeed using the apparatus of dialectical thinking, being able to find the opposites and their relationship. In regard to the understanding of mental or cognitive processes behind this analysis, they are represented by mathematical models in the Figure 1. These models can be interpreted as a closed system described from the operational perspective. However, the dialectical structure depicted there contains development moving toward increasingly complicated levels. It is linked to the construction of new levels that are being created under a similar principle (Veraksa and Zadadaev, 2017).

One could assume that the structural-dialectical approach might not be efficient. Such a conclusion would be justified if application of the approach could be reduced to the statement of the fact of existence of the relations of opposites when characterizing various objects. Yet, the main feature of this method is actually the ability to describe the transformations of material and conceptual objects in relation to the opposites that the individual identified as the structural units. In other words, the structural-dialectical method allows comprehension of the logic of possible transformations or changes of an object and consideration of dialectical logic as the logic of opportunities. This is what constitutes the fundamental difference of structural interpretation of dialectical logic from traditional approaches. A new tool emerges, and it can serve not only for the registration of some occurring events but also for their anticipation. It formed the basis of a research area that came to be known in Russia as “structural-dialectical developmental psychology” (Bayanova, 2013; Veraksa et al., 2013).

With respect to the practical application of the results of each paradigm and line of research, the profound understanding of dialectical cognition as handling evolving content allowed Davydov to build a system of developmental education. It was based on the idea of identifying the contradictions that generate developmental content. If in a traditional school, teaching was based on the transition from the specific to the formation of abstract concepts through gradual generalization, developmental education proposed the opposite way—from a generalized relationship (which characterizes the main contradiction) to the construction of specific meaningful generalizations. This form of practice, however, presents a certain complexity, which is associated with higher requirements for a pedagogical team in charge of developmental training.

Within the framework of the structural-dialectical approach an adult education technology was developed around the “positional learning” model. It is focused on removing the estrangement between a discipline under study and a student’s personality by engaging any content within contexts that are defined by different positions students take: e.g., the perspective of a critic, a poet, an apologist, etc (Veraksa, 1994).

A research area of no less importance is the development of preschool and school education. Since dialectical relations are part of any school subject and activity, they create a fundamental possibility of distinguishing in the knowledge system the meta-subject content accessible at different ages (Krasheninnikov, 2012). According to multiple researches, a distinctive feature of dialectical education is that the structure of preschoolers’ dialectical cognition continues to be actively used at school age and subsequent ages (Shiyan, 2008), rather than being suppressed in the service of avoiding and eliminating contradiction as is advocated in traditional education.

## Psychotherapy Research as an Example

To illustrate the differences among psychological inquiries founded on universalistic, relativistic, and dialectical thinking, we consider psychotherapy as an area of practice, research, and psychological curriculum. *Schools-cum-approaches* to psychotherapy initially developed more or less as separate, independent communities in the 20th century, each teaching approaches to theory and practice founded on its own assumptions, with little compatibility among them and little concern with their compatibility. Then, following what researchers following dialectical approaches expect, encounters among separate communities occurred, creating conflicts or issues to be resolved among them. Then, researchers taking a more macroscopic view of the subject matter differentiated major approaches which they referred to as psychodynamic, behavioral, existential-humanistic, cognitive, and systemic. Most of these researchers adopted a universalistic approach, within which they asked the question “which theory is most valid and which practice is most effective?,” with the assumption that a straightforward methodology could be used to gather evidence that would satisfactorily provide the answers to these questions. Meanwhile, others instead contributed to a proliferation of different psychotherapies derived from clarifying different variations within these categories as well as from combining components across categories. But a general universalist zeitgeist led the vast majority of developers of the various “types” of therapy with different names to believe that they would need to gather evidence that demonstrated their theoretical and technical superiority to all of the other variants. There was little effort to articulate the historical conditions that brought these thriving communities of practitioners into conflict.

As predicted by hypothesis of Rosenzweig (1936) that claimed the existence of implicit common factors among all the different psychotherapies, only small and nonsignificant differences were found in effectiveness studies of various extant therapeutic approaches (Luborsky, et al., 1999, 2006). Some researchers and practitioners claimed these studies implied that most important for psychotherapy success would be to do “psychotherapy well” (i.e., following adequately one psychotherapy approach will ensure success, regardless of which one chooses to follow). We see this as adopting a largely relativistic approach to psychotherapy practice, research, and curriculum. Further in line with the ascendance of relativistic thought in this area, some therapeutic approaches to psychotherapy (e.g., narrative, constructivist) were founded on explicitly relativistic assumptions (e.g., every psychotherapy client is unique, and every psychotherapy process is unique, so searching for commonalities or general laws of psychotherapy is pointless).

Others facing the common factors hypothesis and psychotherapy challenges, in what could be seen as a dialectical approach to inquiry, espoused the hope for processes of psychotherapy integration to make therapy as effective and efficient as possible, considering both common and theory/technique-specific factors. These integration attempts followed different avenues (Kozarić-Kovacić, 2008; Castonguay et al., 2015): theoretical integration (i.e., creating one differentiated and integrated theoretical approach integrating the perspectives of communities in conflict), technical eclecticism (i.e., therapists being trained to be willing and able to implement effective techniques and elements from different communities), assimilative integration (i.e., using a single theoretical model but integrating ideas and techniques from other communities to overcome limits of effectiveness of one’s model), and common factors approaches (i.e., focusing on common factors or processes within all psychotherapy communities). These attempts to integrate various communities’ perspectives into a single theory/approach that would unite a wide range of theoretical and technical components of psychotherapy practice resulted, ironically, in a further proliferation of integrative theories of psychotherapy during 1980’s and 1990s’ decades. Garfield and Bergin (1994) already documented the existence of over 400 varieties of psychotherapy approaches varying according to their theoretical model, format (i.e., individual, family, and group), brief or long therapies, mental disorder type, and all sorts of combination of different elements. Consequently, what could be seen as an attempt to achieve a synthesis by adopting a dialectical perspective, led in many cases to a return to a universalistic perspective of trying to find which integrative approaches would most increase effectiveness and efficiency for all cases! In our view, while the advocacy of integration was admirable, it was limited by the failure of the approach to be fully dialectical. It treated psychotherapy debates as isolated rather than occurring in the context of socio-economic factors leading to, for example, financial and status competition among advocates of various approaches, the increased role of insurance companies as supporters and therefore gate-keepers of psychotherapy, the need of a more educated group of consumers for protection from exploitation, etc.

Nevertheless, the integrative movement has continued to expand, representing a dialectical attitude of differentiating approaches as useful within specific social and historical contexts, while also integrating approaches to transcend limits of context. Approaches within that movement ask therapists to be responsive and flexible to patients’ needs, taking into account their differences in contexts, cultural lessons learned, and problems (Zarbo et al., 2016). Furthermore, therapists have to recognize that patients’ needs evolve across time and situations; to understand the importance of maintaining and adapting therapeutic alliances across these evolutions over time; and to understand commonalities as well as differences in *developmental process* across psychotherapy cases, both with respect to not only clients’ repertoires but also the alliances themselves among therapists and clients (Basseches and Mascolo, 2010). Viewing psychotherapy cases as developmental processes implies that clients’ development should be fostered, tracked, and evaluated in a process that considers not only the clients’ adaptive challenges but also the adaptive challenges of all the others whom the clients’ actions affect. A dialectical approach to practice and theory clearly demands a psychotherapy curriculum that prepares students to think dialectically about the complex set of interrelated phenomena that psychotherapy comprises, including to recognize the conflicts and potential synthetic resolutions that exist among those phenomena.

But why is dialectical thinking really needed for a post-modern inquiry into psychotherapy? The research briefly summarized before can be puzzling. It seems this field is going in circles between searching for a single common approach that fits all (universalistic formal analysis), and failing on that attempt, to a relativistic perspective that affirms uniqueness of cases and diversity of approaches, while undermining any attempt to provide therapists with avenues to guidance in making the moment-by-moment choices that are needed and to provide the field with any pathways for improving overall interventions and outcomes. Several factors contribute to this “going in circles effect.” One aspect is the failure to emphasize that while there are operative “psychotherapy delivery systems” in most cultures, there are also environmental systems outside those systems which interact with the psychotherapy delivery systems. Such extra-therapeutic systems include client support systems (Drisko, 2004), which may be more or less adequate, as well as economical and sociological systems which influence the contexts and possibilities for psychotherapy. A dialectical approach allows going beyond the dichotomy of nomothetic vs. ideographic approaches to psychotherapy. Development in psychotherapy can be viewed as a movement *through* forms occurring due to some combination of the conflicts and conflict resolution activity within the constitutive and interactive relationships of clients and therapists, as well as within each of the parties’ interactive and constitutive relationships with their environments. Thus, every therapy relationship is a dialectic, and the model of dialectic can be used to track the patterns and challenges within that relationship. The fact that in any psychotherapy process we are dealing with constant interaction within the therapy context and outside of it, requires a dialectic analysis.

In practical terms, we need to maintain awareness of the boundary conditions to which the scope of the value of formal analyses in psychotherapy is limited and include the case-specific context in a way that idiosyncrasies that violate those boundary conditions can be part of the dialectical analyses of psychotherapies. As we have seen in psychotherapy research, when this awareness is not taken into account we end as Vygotsky (1982) forewarned: following “other paths will inevitably lead to scholastic, verbal constructions, to dissolving dialectics in questionnaires and tests, to reasoning about things on the bases of external, casual, secondary features, to losing any objective criteria, to trying to negate any historical trend in the development of psychology.”

Above, we cited the book “*psychotherapy as a developmental process*” of Basseches and Mascolo (2010) in which a dialectical method for assessing micro-developments in therapy across all psychotherapeutic approaches was proposed. The proposal offers a useful framework in differentiating three fundamentally different types of resources that therapists’ actions within the therapy relationship can provide to the therapeutic process, regardless of what guiding model therapists are following and what therapeutic techniques they are employing. The proposal also claims, and has supported this claim with case studies, that the three different types of resource must be integrated for a case of psychotherapy to be successful in some way. Each resource, in its own way, fosters the emergence and exploration of conflict, and the authors’ present a method for tracking utterance by utterance in verbatim dialog the steps that either lead to successful resolutions of conflicts or that leave them unresolved. Unfortunately, this research method is very labor-intensive which limits its practical utility. However, its implied guideline for clinical practice is valuable: To be aware of developmental processes on a moment-to-moment basis, regardless of whether one is following one or many therapeutic and/or technical approaches. What is still needed from a dialectical approach to psychotherapy are tools for clinicians to use to look at their practice at all levels of therapy (short, medium, and long term) including emerging conflicts and resolutions as they develop therapeutic relationships and work with various common factors, techniques, types of clinical problems, and contextual factors. Advances with this proposal or further different proposals will have to ensure ways of integrating nomothetical proposals with a clear map of how to navigate the ideographic aspects of each case, not forgetting that the central aspect of therapy is the transformational process! However, this requires clinicians learning to think dialectically, for which we hope a post-modern psychology will provide a supportive context. Neither a modernist/universalistic nor a relativistic psychology can offer therapists and students of therapy such important tools.

## Discussion

Returning to our introduction, we stated our view that without an expanded view of rational inquiry that offers a model for the construction of more intersubjective, epistemically adequate understandings over time, post-modern inquiry would be limited to the simple accumulations of descriptions of various subjective interpretations of phenomena.

Regarding the form of rational inquiry that may be at the core of modernist inquiry, all of the work on dialectical thinking that we have described recognized that this form of rationality was insufficient by itself. The Vygotskian tradition started with the content of ideas, studied historically. It described in such studies the appearance of contradictory ideas, followed by the appearance of synthetic ideas that resolved the contradictions, only to themselves later be contradicted as natural and social environments changed. It was presumed that through instruction using such historical analysis of content, students would learn to “think dialectically.” Veraksa’s “structural-dialectical developmental psychology” started with the observation that throughout ontogeny, encounters with relations of opposition and contradiction occurred, and that cognitive operations on relationships of opposition and contradiction (recognizing contradiction and transformation) were as essential for coping with both the material world and the conceptual world as operations (such as those described by Piaget) for creating, recognizing, and maintaining stability. It recognized that most educational and other socializing systems tended to privilege the creation and maintenance of fixed order over the appreciation of contradiction and the development of the ability to recognize it and to deal with it in creative and transformative ways. This led to research on questions of how early in child development could the use of dialectical operations be recognized, and how the tendency to suppress the development of this kind of thinking in favor of thinking which identifies, creates and maintains order, could be counteracted educationally.

The focus of Basseches (1984) was on the differences between the most powerful system of creating order described by Inhelder and Piaget (1958)—formal operational thought, and the most complex form of dialectical thinking (now sometimes referred to as “scientific dialectics”) that he found in a wide range of contents of intellectual history, including Piaget’s own account of the process of ontogenesis of the “organ” of intelligence. Basseches saw the capacities for such thinking as rooted in different models or forms of organization that were constructed over the course of cognitive ontogenesis. He proposed that the model underlying formal operational thought (and perhaps “modernist” inquiry) was a model of a “closed-system of lawful relationships.” He contrasted this with a proposed model underlying dialectical thinking of open self-transforming systems in interaction with each other. He claimed that dialectical thinking represented a form of thinking more developed than formal operational thinking because the idea of dialectics required the understanding of a system and represented a differentiation of the concept of a closed system from that which was beyond the limits or boundary conditions of a closed system. Dialectical thinking thus included the capacities to use both closed system and open system models. Basseches’ proposal paralleled Piaget’s argument for why each of his stages represented development beyond the previous one and why each previous stage was a necessary but not sufficient condition for the subsequent stage. It could therefore be seen both as a critique of the limits of Piaget’s project and an extension of that project beyond those limits.

In our view, the study of post-formal dialectical thinking is of great importance because it includes the power of formal analyses, while at the same time having the power to transcend the limitations of closed-system analyses.^4^ Describing and identifying such thinking has been the focus of the work of Basseches and his colleagues. Putting neo-Piagetian and neo-Vygotskian streams of research together, we can ask the following question: how does identifying and promoting the development of dialectical thinking in various periods of childhood^5^ affect the processes and the likelihood of individuals becoming capable in adulthood of creating dialectical analyses, organized by the idea of dialectic, as articulated by Basseches? With sufficient resources a long-term longitudinal study could be conducted that would begin to answer such questions.

Thus, we propose that the study of dialectical thinking be an effort to study the development of dialectical thinking capacities based on (1) acknowledgment of the value of efforts to organize human actions and observations, when contradictions are inevitably discovered and encountered; while at the same time and (2) treating specific organizations created as moments in dialectical processes, and not as fixed unchangeable laws of nature or of human activity.

We articulated our view in our first paragraph that the transition from a modern psychology to an adequate post-modern psychology depends on dialectical thinking. We can end by expanding that articulation in the light of the foregoing. We see modernist inquiry as in some way analogous, if not equivalent, to inquiry aimed at discovering and articulating fixed, lawful regularities undergirded by the structures of formal operational thought. Consistent with Riegel’s (1973) argument, to cling tightly to modernist theories and interpretations of data would be to contribute to the phenomenon he referred to as “alienated thinking” (or “alienated knowing”). On the other hand, to simply reject the products of modern inquiry would be analogous to the devaluing of moments of creating organizing structures when faced with contradictory, opposite, or conflicting actions and observations. This would deny both the value of forms of stability for human life and the importance of bringing together different subjectivities to create intersubjectivities. Doing so would not create an adequate replacement for modern science and inquiry. As an alternative, we propose a post-modern psychology that understands inquiry as composed of temporary moments which represent steps in ongoing processes. Some steps entail finding contradictions between structures and that which is outside of, other than, or unstable within, such structures. Other steps entail transforming understandings to more complex, differentiated and integrated ones by conceptualizing the relationship between what is well-organized within a structure, and that which lies beyond or stands against its organizing power. We hope not only to have outlined a path for future study of dialectical thinking, but also to have implied a pathway for the development of post-modern psychology.

## Author Contributions

All authors listed have made a substantial, direct, and intellectual contribution to the work and approved it for publication.

## Funding

The research was supported by the RSF grant 19-18-00521-П.

## Conflict of Interest

The authors declare that the research was conducted in the absence of any commercial or financial relationships that could be construed as a potential conflict of interest.

## Publisher’s Note

All claims expressed in this article are solely those of the authors and do not necessarily represent those of their affiliated organizations, or those of the publisher, the editors and the reviewers. Any product that may be evaluated in this article, or claim that may be made by its manufacturer, is not guaranteed or endorsed by the publisher.

## Footnotes

^1^See (Basseches, 1984, p. 10–12).

^2^Kegan (1982) cites Wells (1972) as documenting a transformation toward more dialectical approaches in nearly every social and natural science during the last 150 years.

^3^The presentation of the 24 schemata abstracted from various writings reflecting dialectical world-outlooks, then supplemented and modified based on pilot interviews, is beyond the scope of this article. The readers that want to go deeper on this subject should read the book “dialectical thinking and adult development” by Michael Basseches. Based on his data set, Chapter 4 of the book describes the overall organization of the 24 dialectical schemata and provides several examples of each dialectical schema drawn from his interviews with research subjects.

^4^More generally, we would say that within the dialectics of cognitive ontogeny, those moments of creating order out of contradiction, as well as the moments of recognition of contradictions that challenge existing orderings, are equally important and mutually dependent and complementary. And this is equally true for the development of knowledge at the phylogenetic level as well.

^5^While counteracting the alienating effect of education that focuses on apprehending structures—those objectified (perhaps even fetishized—oh those correct answers on tests that translate into grades on report cards feel good!) products of organizing contradictory experience—at the expense of encouraging the process of dealing creatively and constructively with contradiction.

## References

[ref1] Adorno T. W. Horkheimer M. (1979). Dialectic of Enlightenment. London: NLB.

[ref001] Allport G. W. (1937). Personality: A psychological interpretation. New York: Holt, Rinehart, and Winston.

[ref2] Andreev I. D. (1985). Dialectical Logic. Мoscow: Visshaya Shkola.

[ref3] Basseches M. (1978). Beyond closed-system problem-solving: A study of metasystematic aspects of mature thought. Doctoral Dissertation, Harvard University, University Microfilms, Ann Arbor, MI, USA.

[ref4] Basseches M. (1980). Dialectical schemata: a framework for the empirical study of the development of dialectical thinking. Hum. Dev. 23, 400–421. doi: 10.1159/000272600

[ref5] Basseches M. (1984). Dialectical Thinking and Adult Development. Norwood, NJ, USA: Ablex.

[ref7] Basseches M. Mascolo M. (2010). Psychotherapy as a Developmental Process. New York: Routledge.

[ref8] Bayanova L. F. (2013). Vygotsky’s hamlet: the dialectic method and personality psychology. Psychol. Rus. State Art 5, 35–42. doi: 10.11621/pir.2013.0103

[ref9] Castonguay L. G. Eubanks C. F. Goldfried M. R. Muran J. C. Lutz W. (2015). Research on psychotherapy integration: building on the past, looking to the future. Psychother. Res. 25, 365–382. doi: 10.1080/10503307.2015.1014010, PMID: 25800531

[ref10] Chan S. F. (2000). Formal logic and dialectical thinking are not incongruent. Am. Psychol. 55, 1063–1064. doi: 10.1037/0003-066X.55.9.1063, PMID: 11036714

[ref002] Chomsky M. (1957). Syntactic Structures. The Hague: Mounton.

[ref11] Davydov V. V. (1972). Means of Generalization in Education. Мoscow: Pedagogika.

[ref12] Drisko J. W. (2004). Common factors in psychotherapy outcome: meta-analytic findings and their implications for practice and research. Fam. Soc. 85, 81–90. doi: 10.1606/1044-3894.239

[ref13] Feyerabend P. (1975). Against Method: An Outline of an Anarchistic Theory of Knowledge. London: NLB.

[ref14] Flavell J. (1967). Genetic Psychology of Jean Piaget. Moscow: Prosveshenie.

[ref15] Garfield S. Bergin A. (1994). “Introduction and historical overview,” in Handbook of Psychotherapy and Behaviour Change. eds. Bergin A. Garfield S. (Chichester: Wiley), 3–18.

[ref16] Hamamura T. Heine S. J. Paulhus D. L. (2008). Cultural differences in response styles: the role of dialectical thinking. Personal. Individ. Differ. 44, 932–942. doi: 10.1016/j.paid.2007.10.034

[ref17] Hegel G. W. F. (1821/1965). The Philosophy of Right. Chicago: Encyclopedia Britannica.

[ref18] Ho D. Y. F. (2000). Dialectical thinking: neither eastern nor western. Am. Psychol. 55, 1064–1065. doi: 10.1037/0003-066X.55.9.1064, PMID: 11036715

[ref19] Horz H. Poltz H. Parthey H. Rosenberg U. Wessel K. (1980). Philosophical Problems in Physical Science. Minneapolis, MN: Marxist Educational Press.

[ref20] Ilyenkov E. V. (1974). Dialectical Logic. Мoscow: Politizdat.

[ref21] Ilyenkov E. V. (1979). Dialectical Contradiction. Мoscow: Politizdat.

[ref22] Inhelder B. Piaget J. (1958). The Growth of Logical Thinking From Childhood to Adolescence. New York: Basic Books.

[ref23] Jameson F. (1971). Marxism and form: 20th century dialectical theories of literature. Princeton, NJ: Princeton University Press.

[ref24] Jay M. (1973). The Dialectical Imagination. Boston: Little Brown and Company.

[ref25] Kegan R. (1982). The Evolving Self: Problem and Process in Human Development. Cambridge, MA, USA: Harvard University Press.

[ref26] Kilminster R. (1979). Praxis and Method: A Sociological Dialogue With Lukacs, Gramsky, and the Early Frankfurt School. London: Routledge & Kegan Paul.

[ref27] Kodric-Brown A. (1986). Satellites and sneakers: opportunistic male breeding tactics in pupfish (*Cyprinodon pecosensis*). Behav. Ecol. Sociobiol. 19, 425–432. doi: 10.1007/BF00300545

[ref28] Kopnin P. V. (1973). Dialectics as Logic and Theory of Mind. Мoscow: Nauka.

[ref29] Kozarić-Kovacić D. (2008). Integrative psychotherapy. Psychiatr. Danub. 20, 352–363. PMID: 18827763

[ref30] Krasheninnikov Е. Е. (2012). Development of Cognitive Abilities of Preschoolers. For Work With Children 4–7 Years Old. Мoscow: Mozaika-Sintez.

[ref31] Kuhn T. S. (1970). The Structure of Scientific Revolutions. Chicago: University of Chicago Press.

[ref003] Leontiev A. N. (1983). Selected Psychological Works: In 2 vols. Vol. II. Moscow: Pedagogy.

[ref32] Luborsky L. Diguer L. Luborsky E. Schmidt K. A. (1999). “The efficacy of dynamic versus other psychotherapies: is it true that “everyone has won and all must have prizes?”—an update,” in Psychotherapy Indications and Outcomes. ed. Janowsky D. S. (Washington, DC: American Psychiatric Press), 3–22.

[ref33] Luborsky L. Rosenthal R. Diguer L. Andrusyna T. P. Berman J. S. Levitt J. T.. (2006). The dodo bird verdict is alive and well-mostly. Clin. Psychol. Sci. Pract. 9, 2–12. doi: 10.1093/clipsy.9.1.2

[ref004] Maltsev V. I. (1964). Essay on dialectical logic. Moscow: Moscow University Press.

[ref34] Mandel E. (1973). An Introduction to Marxist Economic Theory. New York: Pathfinder.

[ref35] Marx K. (1844/1967). in Economic and Philosophical Manuscripts in Writings of the Young Marx on Philosophy and Society. eds. Easton L. D. Guddat K. H. (Garden City, New York: Doubleday & Company)

[ref36] Marx K. Engels F. (1848/1955). The Communist Manifesto. New York: Appleton-Century Crofts.

[ref005] Mead M. (1928). Coming of age in Samoa. New York: Morrow.

[ref37] Ng A. H. Hynie M. (2016). Naïve dialecticism and indecisiveness: mediating mechanism and downstream consequences. J. Cross-Cult. Psychol. 47, 263–276. doi: 10.1177/0022022115613861

[ref38] Peng K. Nisbett R. E. (1999). Culture, dialectics, and reasoning about contradiction. Am. Psychol. 54, 741–754. doi: 10.1037/0003-066X.54.9.741

[ref39] Peng K. Spencer-Rodgers J. Nian Z. (2006). “Naïve dialecticism and the Tao of Chinese thought,” in Indigenous and Cultural Psychology: Understanding People in Context. eds. Kim U. Yang K.-S. Hwang K.-K. (Springer Science + Business Media).

[ref40] Piaget J. (1952). The Origins of Intelligence in Children. New York: Norton.

[ref41] Piaget J. (1954). The Construction of Reality in the Child. New York: Basic Books.

[ref42] Piaget J. (1967). Six psychological studies. New York: Random House.

[ref43] Piaget J. (1970). Structuralism. New York: Basic Books.

[ref44] Piaget J. (1974). Recherches sur la Contradiction. Etudes D’Epistemologie Genetique. Paris: P.U.F. 31, 32.

[ref45] Piaget J. (1977). Recherches Sur l’Abstraction Reflechissante. Etudes d’Epistemologie Genetique. Paris: P.U.F. 34, 35.

[ref46] Popper K. R. (1959). The Logic of Scientific Discovery. New York: Basic Books.

[ref006] Porus V. N. (1979). Dialectical contradiction. Moscow: Politizdat.

[ref47] Provine W. (1971). The Origins of Theoretical Population Genetics. Chicago, IL: University of Chicago Press.

[ref007] Quora (2014). First answer in Google.com search for “modern vs. postmodern” by Roberta Pearce in Quora. Available at: https://www.quora.com/What-is-the-difference-between-modern-and-postmodern-art?top_ans=5280438

[ref48] Reichenbach H. (1938). Experience and Prediction. Chicago, IL: University of Chicago Press.

[ref49] Riegel K. F. (1973). Dialectic operations: the final period of cognitive development. Hum. Dev. 16, 346–370. doi: 10.1159/000271287, PMID: 4591158

[ref50] Rosenzweig S. (1936). Some implicit common factors in diverse methods of psychotherapy. Am. J. Orthop. 6, 412–415. doi: 10.1111/j.1939-0025.1936.tb05248.x

[ref51] Rubinstein S. L. (1957). Being and Consciousness. Мoscow: Izdatelstvo АN USSR.

[ref52] Shiyan О. А. (2008). Developing education in university: dialectical structure of the educational course as a source of student development. Psychol. Sci. Educ. 2, 9–17. doi: 10.17759/pse

[ref53] Smith A. (1776/1937). An Inquiry Into the Nature and Causes of the Wealth of Nations. New York: Modern Library.

[ref54] Spencer-Rodgers J. Williams M. J. Peng K. (2010). Cultural differences in expectations of change and tolerance for contradiction: a decade of empirical research. Personal. Soc. Psychol. Rev. 14, 296–312. doi: 10.1177/1088868310362982, PMID: 20435801

[ref55] Veraksa N. (1981). Peculiarities of the transformation of conflicting problem situations by preschool children. Voptosy Psikhol. 3, 123–127.

[ref56] Veraksa N. (1994). The model of standpoint teaching of students. Voptosy Psikhol. 3, 122–129.

[ref57] Veraksa N. (2006). Dialectical Thinking. Ufa: Vagant.

[ref58] Veraksa N. Belolutskaya A. Vorobyeva I. Krasheninnikov E. Rachkova E. Shiyan I.. (2013). Structural dialectical approach in psychology: problems and research results. Psychol. Rus. State Art 6, 65–77. doi: 10.11621/pir.2013.0206

[ref59] Veraksa N. Zadadaev S. (2012). Dialectical thinking and W-measure of the development of two-dimensional dialectical structure. Psychol. Sci. 15, 57–86.

[ref60] Veraksa N. Zadadaev S. (2017). Foundations of structural dialectics. Herald of the MCU 3, 8–25.

[ref62] Vygotsky L. S. (1982). Collected Works in 6 1. Moscow: Pedagogika.

[ref63] Vygotsky L. S. (1983). Collected Works in 6 5. Moscow: Pedagogika.

[ref64] Wells H. K. (1972). Alienation and dialectical logic. Kansas J. Sociol. 3, 1.

[ref65] Zarbo C. Tasca G. A. Cattafi F. Compare A. (2016). Integrative psychotherapy works. Front. Psychol. 6:2021. doi: 10.3389/fpsyg.2015.02021, PMID: 26793143 PMC4707273

[ref66] Zheng W. Yu A. Li D. Fang P. Peng K. (2021). Cultural differences in mixed emotions: the role of dialectical thinking. Front. Psychol. 11:538793. doi: 10.3389/fpsyg.2020.538793, PMID: 33505326 PMC7830092

